# Rodent Ulcer

**Published:** 1908-02-22

**Authors:** 


### Points in Surgery.

### RODENT ULCER.

Rodent ulcer is a very peculiar disease. It is undoubtedly malignant, and yet it differs from genuine malignant disease in nearly all its characteristics. Thus it does not infect lymphatic glands, and it does not give rise to metastatic deposits in other parts of the body in the way that carcinoma does. It is generally single, but may be multiple, whereas in carcinoma proper more than one original focus of the disease is rarely if ever found in the same individual. One sometimes hears of instances in which carcinomatous foci were found simultaneously in both breasts, or in the ovary and the breast at the same time. But these cases are most reasonably and logically explained on the hypothesis that one focus really started before the second, which is due to metastatic dissemination, and not by supposing that the two tumours are independent foci of malignant disease. And, lastly, although a rodent ulcer may eventually destroy the life of its possessor, it takes many years to do so, and it is often found that these patients succumb in the end to some quite different intercurrent malady.

Another curious feature about rodent ulcer is that although clinically it must be regarded as the least malignant of all forms of malignant disease, it shows a marked tendency to recur *in situ*, even when it is removed in its early stages, and a wide margin of healthy tissue cut away all round it. The following history of a case seen recently is quite a common one to obtain in this condition. The patient, a man aged sixty-four, had first noticed a small pimple on the right side of his cheek some eight years previously. He took no notice of it for four or five years until it grew to the size of, and looked like, a ripe red currant. He then sought advice, and the tumour was removed. He was then quite well until six months ago, when he noticed the pimple beginning to grow again in the same situation. Recently it has, to use his own phrase, "become ripe and burst" several times, healing over after each occasion.

The case illustrates several points. First the slow growth of the disease; it took five years to increase from a pimple to the size of a ripe red currant, and after all that time gave rise to no symptoms. Secondly the tendency to recur *in situ*, in spite of a removal that was apparently complete; that is to say, the excised tissue, when examined miscroscopically, showed a wide margin of normal structures all round the growth. Moreover, the commencement of the disease was characteristic: patients will nearly all tell you that it started as a pimple. That is to say, it begins as a solid growth, a papule or a flat-topped excrescence, which is often coloured a deep red or mulberry colour. There is sometimes a hyperæmic reaction round the growth, but this is not constant. It is only later, in some cases, as in this one, after many years, that ulceration makes its appearance, and this patient's expressive phrase that the tumour "became ripe and burst" gives one quite a good idea of the nature of the process. Before ulceration occurs the solid growth tends to spread laterally, and, although it grows slowly, it may have attained quite a considerable size before ulceration occurs. But as soon as it ulcerates it involves the deeper structures progressively, invading at last even the subjacent bone, and cases have been recorded in which even the dura mater has been exposed by this process.

A rodent ulcer usually starts in the skin of the face just below the inner canthus of the eye, but it may attack any part of the skin of the face or neck. In other parts of the body it is excessively rare. When it starts in the neighbourhood of the lip it may be difficult to diagnose from epithelioma in the same situation without a microscopical examination. There is, however, one point which may help to elucidate the diagnosis. Epithelioma nearly always starts in connection with the red margin of lip, while rodent ulcer hardly ever does this. So that if there be a portion of skin uninvolved between the edge of the growth and the margin of the lip, the condition may be confidently said to be a rodent ulcer.

This is only to be expected when one remembers the pathology of rodent ulcer. It is a carcinoma which has its origin in one of the appendages of the skin. Whether it be in the sweat glands or in the sebaceous glands or, as has been more recently suggested, in the deeper layers of the rete Malpighii, is not agreed upon; but at any rate it begins in a structure which is not normally present in the red margin of the lip.

Microscopically a rodent ulcer presents appearances intermediate between those of epithelioma and glandular carcinoma. It consists of cells arranged in alveoli, but these are irregular in shape, being often flattened so as to resemble the downgrowths seen in epithelioma; moreover, the alveoli are not discrete, but run into one another, and the cells lining them are not typical epidermal cells, but are smaller and resemble in type the deeper cells of the epidermus. Cell nests are not found.

The introduction of $x$-rays as a therapeutic measure has revolutionised the treatment of rodent ulcer. Formerly the practice was to excise the ulcer or nodule widely if this were practicable; but if not, to scrape its surface and apply some caustic like zinc paste. But in recent years wonderful results have been obtained by exposure of the ulcer to $x$-rays for a short period daily. A reaction follows, which results in the ulcer healing and clearing up com- the skin which is formed over the site of the ulcer pletely; and, what is more remarkable, it is said that is quite supple, and that practically no scar is left. Radium bromide is said to be more satisfactory still. Recurrences, however, do occur, and when they do they must be treated in the same way. These measures appear to be of little or no avail when once the subjacent bone has become involved. In fact, the prospect of arresting the disease when this stage has been reached is very bad indeed, even by extensive surgical procedures.

